# The Additional Effect of Neurodynamic Slump and Suboccipital Muscle Inhibition to Passive Stretching of the Short Hamstring: A Single-Blind, Randomized Controlled Trial

**DOI:** 10.3390/healthcare12212152

**Published:** 2024-10-29

**Authors:** Hadaya M. Eladl, Olfat Ibrahim Ali, Osama R. Abdelraouf, Zizi M. Ibrahim, Bodor Bin Sheeha, Alaa Mohammed Alabas, Sara H. Alzare, Wafaa Mahmoud Amin

**Affiliations:** 1 Department of Physical Therapy and Health Rehabilitation, College of Applied Medical Science, Jouf University, Sakaka 2014, Saudi Arabia; hd_mos@yahoo.com (H.M.E.); alaa.alabas1996@gmail.com (A.M.A.);; 2 Department of Physical Therapy for Surgery, Faculty of Physical Therapy, Cairo University, Giza 12613, Egypt; 3 Physical Therapy Program, Batterjee Medical College, Jeddah 21442, Saudi Arabia; olfat.sayed@bmc.edu.sa (O.I.A.); pt4.jed@bmc.edu.sa (O.R.A.); 4 Departement of Basic Science for Physical Therapy, Faculty of Physical Therapy, Cairo University, Giza 12613, Egypt; 5 Department of Rehabilitation Sciences, College of Health and Rehabilitation Sciences, Princess Nourah bint Abdulrahman University, P.O. Box 84428, Riyadh 11671, Saudi Arabia; bhbinsheeha@pnu.edu.sa; 6 Department of Physical Therapy, College of Nursing and Health Sciences, Jazan University, Jazan 45142, Saudi Arabia; wafaa_770@yahoo.com; 7 Basic Science Departement for Physical Therapy, Faculty of Physical Therapy, Cairo University, Giza 12613, Egypt

**Keywords:** hamstring flexibility, neurodynamic, suboccipital inhibition, static stretching

## Abstract

Background/Objectives: Hamstring shortening is a significant musculoskeletal condition affecting the posture and mobility of the spine and lower extremities. This study examined the impact of incorporating neurodynamic slump stretch and suboccipital muscle inhibition into passive static stretching on hamstring flexibility in individuals with short hamstrings. Methods: 117 female participants were classified into three groups: the control group, which received passive static stretch of the hamstring muscle; the neurodynamic slump group, which received neurodynamic slump stretch with passive static stretch; and the suboccipital muscle inhibition group, which received suboccipital muscle inhibition with passive static stretch, for three sessions a week, 10 min each, for four weeks. The outcome measures were the popliteal angle test (PAT), straight leg raising (SLR) test, and forward bending test (FBT) at baseline, immediately following the first session and after four weeks. Results: Statistically significant differences were found within groups (*p* < 0.001) for all outcome measures. Between the groups, there was a more significant improvement in the PAT and the SLR tests, favoring the neurodynamic slump and suboccipital muscle inhibition groups in comparison with the passive static stretch group (*p* < 0.001) with no significant difference between the two groups after the first session and at four weeks of treatment. However, the FBT showed no significant differences immediately following the first session or at four weeks of treatment (*p* > 0.05). Conclusions: This study found that incorporating neurodynamic slump stretch and suboccipital muscle inhibition into passive static stretch techniques effectively treats short hamstrings in healthy individuals.

## 1. Introduction

Flexibility is a crucial factor in an individual’s ability to move seamlessly, improving safety and the efficacy of optimal physical activity. Muscle flexibility is a fundamental aspect of human functionality. However, restricted flexibility can increase the risk of musculoskeletal overuse problems and significantly impact an individual’s functional capacity [1].

The hamstring muscles represent a prime example of a muscle group with a greater tendency to shorten [2]. According to reports, the hamstring is the most frequently injured two-joint muscle group in the body, with the muscle’s poor extensibility being the leading cause of injury. The hamstring muscles play a crucial role in controlling the motion of the hip and knee joints [3,4,5], controlling the pelvis and spine, and maintaining postural alignment. Shortening in the hamstring muscles can result in a posterior pelvic tilt, flattening of the lumbar spine [6,7], restricted range of motion (ROM) of hip and knee joints, an abnormal gait pattern [8], low back pain, and anterior patellofemoral pain [9]. Therefore, it is crucial to investigate and improve the hamstring’s flexibility using the most effective techniques.

Poor postural habits and prolonged periods of sitting are the primary causes of hamstring shortening in young, healthy adults [10]. Hamstring shortening has been identified as a prevalent issue among undergraduate university students, primarily due to prolonged periods of sitting, the adoption of crossed-leg postures for several hours, and a lack of physical activity. The prevalence of hamstring shortening has resulted in a decline in performance and quality of life among this demographic [11,12]. Considering these findings, this study focused on college students to develop an effective treatment method for them.

The superficial backline (SBL) is a vital part of myofascial chains and comprises the plantar fascia, the gastrocnemius muscle, the hamstring muscles, the sacrotuberous ligament, and the erector spinae. The SBL efficiently transfers force between the spine, the pelvic area, the lower limbs, and the upper limbs. In the SBL, the fascia and muscle connect, acting as a single link that impacts the whole chain [13]. Therefore, it is essential to emphasize diagnosis and treatment procedures considering the anatomical connection between the fascia and the muscles [13,14].

Several studies have investigated the effectiveness of different stretching techniques in enhancing the flexibility of the hamstring muscle. Passive static stretching (PSS) is the most prevalent localized technique employed as a standalone or with other stretching methods, including neurodynamic stretching exercises, proprioceptive neuromuscular facilitation (PNF) stretching [6,12] and the suboccipital muscle inhibition technique (SMI), which has been employed as a distance technique to enhance hamstring flexibility [15,16]. Most of these studies have concentrated on the immediate effects of localized and distant stretching techniques for the hamstring muscle, with each technique investigated separately.

Liyanage et al. (2024) systematically reviewed the interventions used to treat hamstring tightness in university students. They found that combining PSS and neurodynamic techniques is the most effective, with comparable outcomes to other modalities. Nevertheless, further evidence and investigations are required [12]. Since the hamstring muscles are mechanically connected to the sciatic nerve, neurodynamics may influence the hamstring’s flexibility. Researchers have suggested that applying a neurodynamic slide to the sciatic nerve and its mechanical interactions could improve the immediate and transient flexibility of the hamstring in tight individuals. A neurodynamic slide could be used alone or with a static stretch [17]. The various hamstring stretching techniques have both muscular and neural effects, increasing muscle length and sciatic nerve tension. Both effects are necessary for optimizing the ROM at the hip and knee joints. Stretching techniques generate neural tension through structural differentiation maneuvers such as neck and ankle dorsiflexion [18,19].

Castellote-Caballero et al. (2014) conducted a study that compared the short-term efficacy of neurodynamic sliding to PSS of the hamstring. The results confirmed that neurodynamic sliding had a more significant effect than PSS. The authors recommended that future studies investigate the long-term effects of combining these techniques to identify the most effective modality for improving hamstring flexibility [6]. Neural sliding and stretching exercises are used as part of the treatment to increase the hamstring’s flexibility and decrease the mechanosensitivity of the posterior neural tissues of the lower extremity caused by the decreased flexibility of the hamstring muscle [20,21,22,23]. It has been indicated that both neural sliding and stretching have an immediate positive effect on active knee extension (AKE) angle and functional flexibility of the hamstring in wrestlers and soccer players [22,23].

Researchers studied the effect of myofascial release (MFR) techniques, including suboccipital and plantar fascia, on the flexibility of the hamstring due to the anatomical link between the skeletal muscles and fascia [13]. Joshi et al. (2018) investigated the short-term effects of MFR on both suboccipital and plantar fascia using the passive knee extension angle test and sit-and-reach test to measure hamstring flexibility; results confirmed that adding MFR with PSS significantly improves the flexibility of the hamstring muscles [16].

The SMI technique is well known as a localized manual therapy technique for improving the mobility of the upper cervical spine. However, its functional effect on other distant structures and the mobility of hip and knee joints is under investigation [24,25]. The hamstring and suboccipital muscles are part of a posterior myofascial chain of SBL, with the neural connection of the suboccipital to the dura mater accordingly; increased tension in this region will result in shortening of the hamstring and compression on the peripheral nerve roots of the lower extremity [25]. The muscle fascia release allows for more excellent stretch and reduced hamstring muscle tone [26].

Aparicio et al. (2009) studied the immediate isolated effect of the SMI technique on hamstring flexibility in university students and footballers. The technique modified the muscles’ elasticity [25]. In a study by Jeong et al. (2018), SMI and craniocervical flexion exercise (CCFE) resulted in equal immediate effects on hamstring flexibility and cervical spine mobility in individuals with neck pain [27].

A literature review revealed a paucity of studies comparing distant stretching techniques (SMI) to local-specific stretching techniques (PSS) and neurodynamic stretching for the hamstring muscles. The literature suggests that investigating the combined effect of different stretching techniques on improving hamstring flexibility would be beneficial [12,25,26]. Therefore, the objective of this study was to examine the influence of adding neurodynamic slump versus suboccipital muscle inhibition to the passive static stretch on the popliteal angle test (PAT), straight leg raising test (SLR), and forward bending test (FBT) in participants with short hamstrings.

## 2. Materials and Methods

### 2.1. The Design of This Study

This study utilized a single-blind, randomized controlled trial (RCT) design, adhering to the ethical principles outlined in the Declaration of Helsinki. This study was conducted between February 2023 and April 2024. Participants were randomly assigned into one of three intervention groups, each receiving a different stretching method; the control group received only passive static stretch (PSS) of hamstring muscle; the suboccipital muscle inhibition group (SMI) received suboccipital muscle release in addition to PSS; and the neurodynamic sliding group (NDS) received slump stretch in addition to PSS. The evaluation was carried out pre-intervention to assess baseline measurements, followed by immediate evaluation after the first session and upon completion of the treatment program. Outcome measures included the popliteal angle test (PAT), straight leg raising test (SLR), and forward flexion test (FFT). This study was conducted over a period of four weeks. The primary objective was to compare the effectiveness of the three different stretching methods on PAT, SLR, and FFT.

### 2.2. Randomization

An independent individual used a computer-generated random number table for the randomization process. Randomization was done using Microsoft Excel for Windows software version 13 (Microsoft Corporation, Redmond, WA, USA).To maintain anonymity, the randomization codes were securely placed in consecutively numbered, sealed opaque envelopes. Participants were then randomly assigned to one of three treatment groups. One of the authors, who was not involved in participant recruitment or data collection, did the randomization process to ensure allocation concealment.

### 2.3. Participants

**Figure 1:**
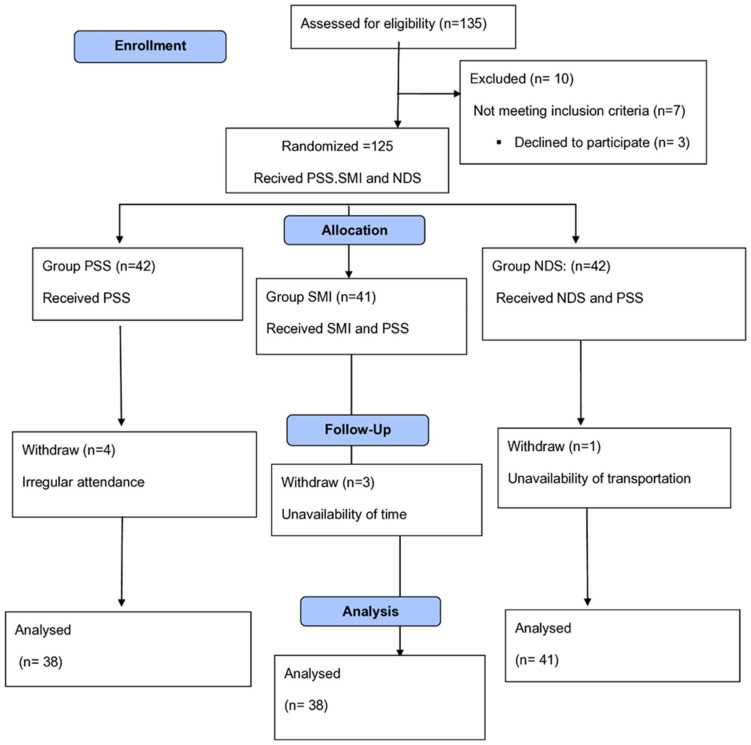
CONSORT flowchart.

One hundred and seventeen collegiate female students volunteered to participate in this study. Participants were assessed for their eligibility to participate in this study. The subjects ranged in age from 18 to 25 years old, and their body mass index (BMI) fell within the range of 18 to 25 kg/m^2^. BMI was calculated using the formula: BMI = weight (kg)/height (m)^2^ [1]. This age range was selected because it represents a period of physical maturation that allows one to focus on functional flexibility without the influence of age-related physiological changes [28]. This study included all participants who met the following criteria: The participants underwent the Popliteal Angle Test (PAT), indicating a tight hamstring with an active knee extension angle e exceeding 15° [16], and a straight leg raising (SLR) with less than 70°of hip flexion, as these criteria represent clinically significant markers of hamstring tightness and essential indicators for assessing flexibility interventions [29]. Individuals who met any of the following criteria were excluded from participation: having experienced a hamstring trauma within the previous year, regularly engaging in lower extremity stretching exercises, having a history of a neck whiplash injury, showing symptoms related to the cervical spine, having recently suffered a fracture in any body area, participants undergoing antidepressants or muscle relaxants, having a history of growth milestones, or having a history of orthopedic or neurological conditions, having a diagnosis of a herniated disc, or having experienced low back pain in the last six months [6]. Participants were randomized to one of three treatment groups: the control group received only PSS of hamstring muscle; the SMI group received suboccipital muscle release in addition to PSS; and the NDS received neurodynamic slump stretch in addition to PSS Figure 1.

### 2.4. Ethical Consideration

Before the study’s commencement, all participants received detailed information about the procedures and the study’s aim, including their right to withdraw at any time. Before the study began, all participants were required to provide written informed consent. The study was approved by the ethical committee (P.T.REC/012/003242) and the clinical trial registry (NCT04932707). It was conducted in accordance with CONSORT guidelines.

### 2.5. Sample Size Calculation

The requisite sample size was calculated using the G*Power 3.1.9.7 software developed by the University of Düsseldorf in Düsseldorf, Germany. The effect size was determined to be 0.25 according to the F test, the alpha level was set at 0.05, and 90% power was deemed necessary. Based on the primary outcome measure of PAT, we estimated the total sample size to be 111 participants, 37 participants in each group, and we increased the sample size to 125 to account for the anticipated dropout rate. The final number in each group was 38 in the PSS group, 38 in the SMI group, and 41 in the NDS group.

### 2.6. Outcomes Measure

The popliteal angle test (PAT) was used as the primary outcome measure due to its relevance and reliability in assessing hamstring flexibility. The secondary outcomes were the straight leg raising (SLR) and forward bending test (FBT). These tests offer a comprehensive evaluation of hamstring flexibility and its functional implications. To prevent potential assessment bias, assessors were blinded to the study’s goal and group allocation when evaluating the outcome measures. The outcome measures were evaluated before the intervention, immediately after the first session, and upon completion of the treatment program.

### 2.7. Assessment Procedures

We rigorously evaluated all participants to determine their eligibility for inclusion in the study. We recorded each subject’s weight and height. Measurements were taken on the dominant lower extremity [6], which is the leg used to kick a ball.

#### 2.7.1. Popliteal Angle Test (PAT) or Active Knee Extension Test (AKE)

Popliteal angle test (PAT) or active knee extension test (AKE) is a validated measure of hamstring tightness, with an intra-tester reliability of 0.99, and is particularly sensitive in detecting the restricted range of motion [30]. The knee flexion angle during the PAT was measured using a standard goniometer. The test was conducted in a supine position with the hip and knee joints of the participant flexed to 90 degrees. As a result, the participant extended the knee joint of the tested leg to the greatest extent possible while maintaining a 90-degree hip joint flexion. Subsequently, the goniometer was placed at the lateral epicondyle of the femur, with one arm aligned with the femur and the other with the tibia. The same trained therapist took each measurement to reduce inter-rater variability [1,16,25,31].

#### 2.7.2. Straight Leg Raising Test (SLR)

The SRL test is commonly used in clinical settings to assess hamstring flexibility and nerve mobility, exhibiting excellent inter-rater reliability with a coefficient of 0.95 to 0.96 [32]. The participant was instructed to lie supine and to lift her tested leg as much as possible, maintaining her knee extended until she felt the first sensation of stretch, pain, or stiffness on the posterior aspect of the thigh or when she started to bend her knee. To determine the hip flexion angle, a standard goniometer was placed on the outer aspect of the thigh above the greater trochanter. The fixed arm was parallel to the table, while the other pointed to the lateral femoral condyle and malleolus. At the point of discomfort, we oriented the goniometer-movable arm toward the lateral malleolus [25].

#### 2.7.3. Forward Flexion Test (FFT)

The FFT was used to assess overall spinal and hamstring flexibility, contributing a functional aspect to assessing the interventions’ outcomes. The FFT demonstrated high levels of validity and reliability, with a correlation coefficient of 0.907 to 0.17 [33]. The participant was instructed to perform a stride stand on a footstep, facing the therapist, and then flex the trunk with the hands forward, holding the knee in extension. A measuring tape was used to measure the distance between the distal part of the middle finger, and a ruler on the vertical side of the footstep measured to the nearest tenth of a millimeter [14,25].

### 2.8. Treatment Procedures

A comprehensive stretching regimen was implemented on the short hamstring of the dominant lower leg. The treatment program was conducted for four weeks, with three weekly sessions. Each session lasted 10 min, with a day off in between. The interval between each repetition of the stretching technique during the session was 30 s [33].

All stretching protocols were administered via trained therapists who followed a standardized procedure to ensure participant consistency. Before the study, the physiotherapists underwent specific training to ensure consistency in delivering the interventions across all participants. The force applied during stretching was controlled to maintain uniformity. Therapists applied force until the participant experienced discomfort but not pain, using manual guidelines and visual feedback to ensure the same intensity was applied for each participant. Therapists’ adherence to the protocols was monitored throughout the study, and any deviations were corrected immediately. The physiotherapists who performed treatment procedures were concealed from the participants’ group assignment and the study’s objective to minimize intervention bias. Participant adherence was closely monitored throughout the study by tracking attendance at each session and ensuring compliance with the prescribed stretching interventions. To maintain high levels of motivation and engagement, participants received regular feedback and encouragement from the physiotherapists during each session.

#### 2.8.1. Passive Static Stretching (PSS) of Hamstring Muscle

The three groups of subjects were subjected to passive static stretching exercises. Subsequently, the therapist positioned the subject in a comfortable supine position. The therapist then instructed the subject to flex the hip joint fully while extending the knee to maintain a neutral ankle position. The therapist performed a hamstring stretch on the dominant side, placing one hand on the sole of the foot for dorsiflexion and the other on the knee to maintain elongation. The therapist maintained the stretch for 60 s, followed by a 30-s relaxation period. The therapist repeated this stretching sequence three times, allowing 30 s of rest in between each repetition [34,35].

#### 2.8.2. Neural Dynamic Slump Stretch (NDS)

The subject was positioned in a comfortable, semi-recumbent posture at the edge of the bed, with his feet elevated to prevent contact with the floor. The subject was instructed to assume a military-style upright posture, after which they were asked to slump, flex their neck and knee joints fully extended, and perform active dorsiflexion of the foot [17]. Maintain this position for 60 s, followed by a 30-s rest period before commencing the next repetition [26].

#### 2.8.3. Suboccipital Muscle Inhibition (SMI)

The therapist induced relaxation by placing the individual in a comfortable lying position with closed eyes, thereby preventing the movements of the eyes from increasing the suboccipital muscle tone. The therapist sat at the level of the subject’s head and placed the hands underneath the subject’s head, with their fingers on the posterior arch of the atlas between the occiput and the second cervical vertebrae. The therapist flexed the metacarpophalangeal joints of his hands to 90° and applied stretch pressure to the suboccipital region for two minutes or until the tissue showed signs of relaxation. A 30-s rest period preceded the next repetition [13,25,36].

### 2.9. Statistical Analysis

This study used IBM SPSS version 20 for Windows for statistical analyses. Levene’s and Kolmogorov–Smirnov normality tests were used to check homogeneity and outliers using histograms and box plots. The outcomes measured showed a parametric distribution. Means and standard deviations were calculated for descriptive statistics. The statistical significance level was established at a *p*-value of less than 0.05. A two-way ANCOVA (Analysis of Covariance) test was used to investigate the effect of treatment (passive static stretch, adding neurodynamic slump to passive static stretch, and versus adding suboccipital muscle inhibition to passive static stretch) on PAT, SLR, and FFT at different periods of time, controlling for age and body mass index (BMI) as a covariate. Post hoc tests were applied in cases of significant interaction and significant effect of time. The demographic data of the three groups was compared using a one-way ANOVA.

## 3. Results

### 3.1. Demographic Data

There was no statistically significant difference between groups regarding age, weight, height, and BMI (*p* > 0.05), as shown in Table 1.

Two-way ANCOVA was conducted to compare the effectiveness of adding either neurodynamic or suboccipital stretch to the static stretch while controlling for age and BMI as covariates. There was no significant effect of age on the tested outcomes: PTA (F (1, 112) = 1.110, *p* = 0.294), SLR (F (1, 112) = 0.604, *p* = 0439), or FFT (F (1, 112) = 0.101, *p* = 0.751). Moreover, there were insignificant effects of BMI on PTA (F (1, 112) = 2.235, *p* = 0.138), SLR (F (1, 112) = 6.286, *p* = 0.014), and FFT (F (1, 112) = 1.027, *p* = 0.313); as shown in Table 2.

The two-way ANCOVA revealed a significant interaction between time and group for both tests of PAT, F (2, 112) = 5.332, *p* = 0.006, and SLR, F (2, 112) = 8.685, *p* = 0.001). However, there was no statistically significant interaction regarding FBT (F (2, 112) = 1.776, *p* = 0.174). The partial eta-squared (η^2^) were 0.3, 0.26, and 0.11 for PAT, SLR, and FBT, respectively.

### 3.2. Within Groups Comparisons

**Figure 2:**
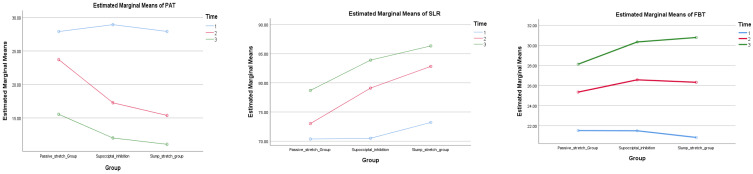
Comparison of PAT, SLR, and FFT before treatment, immediately following the first session, and after four weeks of treatment among the groups.

Post hoc comparisons within groups revealed that the three groups showed a statistically significant decline in the mean PAT and an increase in the mean SLR scores immediately after the initial session and four weeks after the course of treatment (*p* < 0.001). Conversely, there was no significant change in the mean score of FFT, neither after the first session nor after four weeks (*p* > 0.05) as shown in Table 3 and Figure 2.

### 3.3. Between-Group Comparisons

There was no statistically significant difference between the mean values of PAT, SLR, and FFT of all three groups at baseline (*p* = 0.773). However, there was a significant difference among groups post-treatment (<0.001); post hoc comparisons revealed a statistically significant discrepancy between the PSS and NDS groups in terms of PAT, and SLR immediately following the initial session and four weeks of sessions (*p* < 0.001). Similarly, a significant difference was identified between the PSS and SMI groups following the first session (*p* < 0.001) and at the end of the sessions (*p* = 0.002). However, post hoc comparison of the SMI and NDS groups, post-treatment revealed no significant difference regarding PAT and SLR both after the first session (*p* = 0.194, and *p* = 0.018 respectively) and after four weeks (*p* = 0.397 and *p* = 0.19 respectively).

On the other hand, between-group comparisons revealed no statistically significant differences immediately after the first session or at four weeks of treatment (*p* > 0.05). Between-group comparisons for the three outcome measures are demonstrated in Table 3 and Figure 2.

## 4. Discussion

The efficacy of stretching exercises and effective modalities for improving hamstring flexibility remains a topic of ongoing research. However, there is a paucity of data regarding the impact of incorporating suboccipital muscle inhibition, as opposed to the neural dynamic slump stretch, into passive static stretching on the flexibility of the hamstring in subjects with short hamstrings. Further evidence-based practice studies are necessary to provide a basis for clinical decision-making [12]. Based on this, our study compared the combination of passive static stretch with each dynamic neural slump and suboccipital inhibition technique to enhance the health services provided to individuals with shortened hamstrings.

The NDS and SMI groups exhibited the most favorable outcomes. This study’s findings indicate that adding NDS and SMI to PSS is more efficacious in improving hamstring flexibility than PSS alone. Furthermore, the results demonstrate that NDS and SMI effectively treat short hamstrings. The PAT test, SLR, and FFT results demonstrated notable discrepancies within each group. A comparative analysis of the PAT and SLR tests revealed discrepancies between groups. However, the FFT test result was not statistically significant after the initial session or four weeks of treatment.

The improvement observed in the NDS group can be explained by the fact that neurodynamic stretching techniques have been demonstrated to help decrease mechano-sensitivity and increase the mobility of the lower limbs’ posterior neural elements, namely the sciatic, tibial, and common peroneal nerves. This results in a notable enhancement in hamstring flexibility [20,37,38].

The neural dynamic slump stretch results were consistent with previous studies on healthy adults and athletes. A study by Castellote-Caballero et al. (2014) showed that an isolated neural dynamic stretch during the SLR increased hip flexion more than a passive static stretch of the hamstrings did in healthy individuals with short hamstrings [6]. Furthermore, a study by Ahmed and Samhan (2016) evaluated the immediate effects of neural dynamic and static stretching on younger, healthy male adults with short hamstrings. Over the course of five days, they used both techniques for 180 s and measured the results using SRL and the active knee extension (AKE) test. According to the authors’ conclusion, both techniques were effective, with neurodynamic stretching being more effective than static stretching [21]. A similar conclusion was reported by Pratiksha and Bharati [31] and Satkunskiene et al. [39]. Therefore, neurodynamic stretching was proposed as an addition to warm-up routines to enhance hamstring flexibility while maintaining the muscle’s connective tissue stiffness.

To evaluate the effectiveness of various neural mobilization methods, Balcú et al. (2020) and D’souza et al. (2024) compared the immediate effects of neural stretching and neural sliding on the functional flexibility of the hamstring in soccer players and wrestlers. After evaluating sit-and-reach and AKE, the researchers concluded that both techniques were equally effective [22,23].

On the other hand, the significant improvement in hamstrings’ flexibility observed in the SMI group could be attributed to the “sensory theory,” which posits a constraint on the posterior thigh tissues’ flexibility. The sensory theory is employed to elucidate the mechanism by which muscle flexibility is augmented following stretching. Weppler and Magnusson (2010) postulated that stretching techniques result in a mechanical change in the muscle length and modify the individuals’ perception of stretch and pain. This change in perception allows the individual to reach a new endpoint for the limitation of muscle range [40]. Changes in the individuals’ perception of stretch or discomfort during the three flexibility assessments can explain the observed enhancement in hamstring tissue flexibility following the application of the suboccipital muscle inhibition technique [6].

In this context, our results agree with those of previous studies. In their 2009 study, Aparicio and colleagues found improvement in the finger floor distance (FFT), SLR, and popliteal angle (PAT) tests and the pressure algometry of the semimembranosus muscle following the SMI technique in physiotherapy students with tight hamstring muscles [25]. Also, Cho et al. reported that individuals with short hamstrings who received SMI for five minutes showed significant changes in FFT, SLR, and PAT, while those who received a similar duration of self-myofascial release (SMFR) showed a significant increase in SLR only [26]. Furthermore, Prajapati and Shukla (2020) demonstrated the efficacy of the SMI technique on hamstring flexibility in a study involving 52 healthy adults. Over two weeks, the researchers compared the effects of SMI versus static passive stretch, assessing flexibility using the AKE and sit-and-reach tests. They found that both groups exhibited improvements in flexibility, with the Sit-and-Reach (SR) test demonstrating more pronounced outcomes following the SMI application [41].

Another explanation of improvement in the SMI group based on the transmission concept of myofascial force along the posterior chain is supported by the work of Wilke et al., who examined the correlation between the flexibility of the muscles in the lower leg and the cervical spine. The lower limb stretches (LLS) and cervical spine stretch (CSS) were applied to 63 asymptomatic individuals of both sexes. The authors found that stretching the lower limb from a distance improved the cervical range of motion just as quickly as stretching the neck locally [42]. Additionally, Joshi et al. (2018) used static stretching and MFR on the bilateral plantar fascia and suboccipital region and a technique that combined static stretching and MFR on 58 people with tight hamstrings over seven sessions with a two-week follow-up. The results demonstrated that the combination of the remote myofascial release technique with the static hamstring stretch was significantly more effective than static stretching alone in improving flexibility [16].

The present study’s findings regarding comparing the effectiveness of NDS and SMI align with those of Krishna et al. (2021), who concluded that the suboccipital area’s neural dynamics and MFR both effectively enhance the hamstring muscles’ elasticity. However, the SR test for neural mobilization demonstrated a more pronounced effect [43].

Several theorized mechanisms can account for the increased hamstring muscle flexibility in the control group. Firstly, autogenic inhibition, which inhibits muscle stretching while simultaneously relaxing it, leads to increased mobility. Secondly, applying tensile stress to the muscle causes its viscoelastic characteristics to relax gradually, increasing length and ROM constantly. Winter et al. (2004) and Nishikawa et al. (2015) corroborated this, defining passive stretching as the external addition of stretch stimulation to muscle contraction [44,45]. Nevertheless, research indicates that tensile stress, not autogenic inhibition, most likely causes skeletal muscle relaxation. This may provide a potential explanation for the observed enhancement resulting from passive static stretching [6,30,35].

This study’s strength lies in integrating neurodynamic, distant, and local-specific stretching techniques and assessing immediate post-first session and after-four-week outcomes. However, the study has certain limitations. These limitations include the sole measurement of hip and knee joint flexibility without considering other variables like the proprioceptive effect of knee joints and cervical spine flexibility, the use of a single-blind design, and the inclusion of only female subjects. Therefore, we recommend addressing these aspects in future research.

## 5. Conclusions

In various cases of short hamstrings, NDS and SMI must be added to the passive static stretch to maintain hamstring flexibility. This study found a significant positive effect on hamstring flexibility when NDS and SMI were combined with static passive stretch, and there was no difference between the NDS and the SMI technique in patients with short hamstrings.

## Figures and Tables

**Table 1:** Demographic characteristics of the participants.

	PSS G	SMI G	NDS G	*p*-Value
	Mean ± SD	Mean ± SD	Mean ± SD	
Age (years)	22.5 ± 3.4	23.7 ± 4.2	23.1 ± 2.2	0.432
Weight (kg)	160 ± 12.39	57.4 ± 11.9	58.7 ± 12.1	0.518
Height (cm)	157.39 ± 5.27	156.75 ± 4.9	160.1 ± 5.9	0. 098
BMI (kg/m^2)^	22.6 ± 1.88	22.07 ± 2.3	21.59 ± 1.8	0.084

PSSG: passive static stretch group; NDS G: neural dynamic slump stretch group; (SMI): suboccipital muscle inhibition group; SD: standard deviation. *p*-value: probability level. BMI: body mass index.

**Table 2:** Analysis of covariance (ANCOVA).

Source		SS	Df	MS	F-Value	*p*-Value
Intercept	PTA	1572.987	1	1572.987	20.159	<0.001
	SLR	5791.518	1	5791.518	76.589	<0.001
	FFT	647.428	1	647.428	5.464	0.021
Age	PTA	86.587	1	86.587	1.110	0.294
	SLR	45.647	1	45.647	0.604	0.439
	FFT	11.951	1	11.951	0.101	0.751
BMI	PTA	174.424	1	174.424	2.235	0.138
	SLR	475.303	1	475.303	6.286	0.014
	FFT	121.687	1	121.687	1.027	0.313
Group	PTA	1074.793	2	537.397	6.887	0.002
	SLR	2597.046	2	1298.523	17.172	<0.001
	FFT	84.660	2	42.330	0.357	0.700
Error	PTA	8469.233	112	75.618		
	SLR	8739.164	112	78.028		
	FFT	13,270.138	112	118.483		

SS: sum of the squares, df: degree of freedom, MS: mean square, BMI: body mass index, SLR: straight leg raising, PAT: popliteal angle test, FFT: forward flexion test.

**Table 3:** Group comparison of PAT, SLR, and FFT before treatment, immediately following the first session, and at four weeks of treatment.

	PSS Group (n = 38) (M ± SD)	SMI Group (n = 38) (M ± SD)	NDS Group (n = 41) (M ± SD)	Effect Size	*p*-Value (PSS vs. SMI)	*p*-Value (PSS vs. NDS)	*p*-Value (SMI vs. NDS)	*p*-Value
				**PAT**				
Baseline#	27.76 ± 6.2	28.87 ± 8.	28.12 ± 6.2	0.30	0.777	0.634	0.435	0.773
Post I&	23.61 ± 7.0	17.18 ± 6.6	15.56 ± 6.71		<0.001	<0.001	0.194	<0.001
Post II@	15.47 ± 5.97	11.95 ± 4.34	11.2 ± 4.79		0.002	<0.001	0.397	<0.001
MD#-& (95%CI)	4.16 (2.01–6.31)	11.69 (9.54–13.83)	12.56 (10.49–14.63)					
MD#-@ (95%CI)	12.29 (10.11–14.51)	16.92 (14.73–19.12)	16.93 (14.81–19.04)					
*p*-value (baseline vs. Post I)	<0.001	<0.001	<0.001					
*p*-value (baseline vs. Post II)	<0.001	<0.001	<0.001					
*p*-value Post I vs. Post II)	<0.001	<0.001	<0.001					
*p*-value *	<0.001	<0.001	<0.001					
				**SLR**				
Baseline	70.6 ± 6.98	70.55 ± 7.4	72.9 ± 5.04	0.26	0.982	0.105	0.99	0.193
Post I&	73.32 ± 6.24	79.16 ± 7.6	82.49 ± 6.53		<0.001	<0.001	0.018	<0.001
Post II@	78.89 ± 4.43	83.95 ± 5.1	86.12 ± 4.22		<0.001	<0.001	0.019	<0.001
MD#-& (95%CI)	2.68 (0.76–4.6)	8.61 (6.71–10.51)	9.6 (7.75–11.42)					
MD#-@ (95%CI)	8.26 (6.38–10.14)	13.39 (11.52–15.27)	13.2 (11.4–15.03)					
*p*-value (baseline vs. Post I)	0.018	<0.001	<0.001					
*p*-value (baseline vs. Post II)	<0.001	<0.001	<0.001					
*p*-value Post I vs. Post II)	<0.001	<0.001	<0.001					
*p*-value *	<0.001	<0.001	<0.001					
				**FBT**				
Baseline	21.68 ± 6.3	21.42 ± 7.23	20.73 ± 9.17	0.11	0.765	0.961	0.796	0.850
Post I&	25.47 ± 7.16	26.53 ± 7.5	26.24 ± 8.9		0.344	0.349	0.986	0.835
Post II@	28.4 ± 6.36	30.37 ± 6.63	30.51 ± 5.95		0.128	0.084	0.838	0.261
MD (95%CI)	3.8 (2.05–5.53)	5.11 (3.36–6.85)	5.51 (3.84–7.2)					
MD (95%CI)	6.7 (4.2–9.22)	8.95 (6.44–11.46)	9.78 (7.36–12.2)					
*p*-value (baseline vs. Post I)	<0.001	<0.001	<0.001					
*p*-value (baseline vs. Post II)	<0.001	<0.001	<0.001					
*p*-value Post I vs. Post II)	0.003	0.001	<0.001					
*p*-value *	<0.001	<0.001	<0.001					

P: probability level; * Significant at *p* ≤ 0.05; SLR: straight leg raising, PAT: popliteal angle test; FFT: forward flexion test; Post 1: immediately after a session; Post II: after four weeks of treatment; MD#-&: the mean difference between baseline and Post I; MD#-@: the mean difference between baseline and Post II; *p*-value *: within-group significance level; *p*-value: significance level between groups.

## Data Availability

The original contributions presented in the study are included in the article, further inquiries can be directed to the corresponding author.
